# The Book World for Medical Students

**Published:** 1893-09-16

**Authors:** 


### The Book World for Medical Students.

### NEW BOOKS FOR MEDICAL STUDENTS.

There is a good deal of difference of opinion as to the place which text-books ought to hold in medical study. Some teachers deprecate the habit of implicit reliance upon books; others think it good that the student should choose a text-book and thoroughly master it. But nobody seriously proposes to do without text-books altogether; and to judge from the large output on the part of medical publishers, the demand for fresh and reliable works on all the subjects of medical study is not growing less but greater. In regard to text-books of medical science, a curious conservatism exists, which no doubt is quite able to justify itself. It is illustrated in what has taken place with regard to such works as Quain's "Anatomy," and Gray's. These books have been revised, re-edited, practically re-written, not once but many times. The last edition of Quain's "Anatomy" is a different book altogether from the first. Nevertheless, the old name and the old framework are retained; and this course has also been followed with several other standard works.

Medical publishers are wont to show special activity in sending forth books for students as the beginning of the winter session approaches. This season is said not to have been so prolific as usual; nevertheless, the number of recent and forthcoming books for medical students is large. The list which is subjoined contains only the names of new books. Most of them have already been issued; but those that have not will, with hardly any exceptions, be ready by the first or second of October. As to the old and well-tried works, the student may safely rely upon the experience of senior students or the advice of his teacher; but a list of some recent books issued by the leading publishers may not be without its uses. The books are grouped according to subject.

Biology—including Botany and Natural History.—"Elementary Text Book of Biology," by Professor Ainsworth Davis (Griffin); "Text Book of Elementary Biology," by Dr. H. J. Campbell (Swan Sonnenschein); "Introduction to the Study of the Amphioxus," by Professor Hatschek, Vienna (Swan Sonnenschein); "The Student's Botany," by Professor Vines (Swan Sonnenschein); "Vertebrate Embryology," by Professor Milnes Marshall (Smith, Elder, and Co.)

Chemistry and Chemical Physiology.—"A Text Book of the Physiological Chemistry of the Animal Body," by Professor Gamgee (Macmillan); "Essentials of Chemical Physiology," by Professor W. D. Halliburton, King's College (Longmans); "A Course of Qualitative Chemical Analysis," by W. G. Valentin, F.C.S., eighth edition, edited by Dr. W. R. Hodgkinson (J. and A. Churchill); "Attfield's Chemistry," new edition (Gurney and Jackson).

Anatomy.—Under this head special mention has to be made of the new edition of "Quain's Anatomy," which is being edited by Professors Schäfer and Thane, of University College, of which the major portion has been issued (Longmans); a new edition of "Heath's Practical Anatomy," edited by Mr. William Anderson, St. Thomas's Hospital, has been issued (J. and A. Churchill); vol. i. of "A Manual of Practical Anatomy," by Professor Cunningham, Trinity College Dublin, will be ready on October 2nd (Young Pentland); a translation of "Methods of Histological Research," by Dr. C. V. Kahlden is announced by Messrs. Macmillan.

Physiology.—A new edition, the fourth, of Landois' Physiology translated, with annotations and additions, by Professor Stirling, of Owen's College, Manchester, has lately been issued by Griffin and Co. The same firm has published "The Physiologists' Note Book," by Dr. Alexander Hill—a work of great value to students.

Public Health.—There has been much activity of late in producing text books of public health and

THE BOOK WORLD FOR MEDICAL STUDENTS—*continued.*

bacteriology. Recent and forthcoming works on public health are: "A Handbook of Public Health and Demography," by Edward F. Willoughby, M.B. (Macmillan); "Dwelling Houses: Their Sanitary Construction and Arrangements," by Professor Corfield, University College (Lewis); "Public Health Laboratory Work," by Henry R. Kenwood (Lewis's Practical series); "Methods of Practical Hygiene," by Professor K. B. Lehmann, translated by W. Crookes, F.R.S. (Kegan Paul).

Pathology.—Vol. II. of "A Text Book of Pathology," by Professor Hamilton, Aberdeen, is announced by Messrs. Macmillan; "Lectures on the Comparative Pathology of Inflammation," by Elias Metchnikoff, of the Pasteur Institute, translated from the French, has just been issued (Kegan Paul).

Bacteriology.—"Elements of Bacteriology for Practitioners and Students," by Dr. S. L. Schenk, Vienna, translated by W. R. Dawson, M.D. (Longmans); "Practical Bacteriology," by Dr. Migula (Swan Sonnenschein).

Surgery.—"Surgery: Its Theory and Practice," by W. Walsham, F.R.S., fourth edition (Churchill's Student's Guide series). "Tumours, Innocent and Malignant: Their Clinical Character and Appropriate Treatment," by J. Bland Sutton, F.R.C S. (Cassell and Co.) "Diseases of the Joints and Spine," by Howard Marsh, F.R.C.S. (Cassell and Co.); "Diseases of the Rectum and Anus," by Charles B. Ball, F.R.C.S, new edition (Cassell and Co.); "Surgical Handbook," by Alexander Miles, M.D. (Scientific Press).

Ophthalmology.—"Text Book of Ophthalmology," translated from the German of Professor E. Fuchs Vienna (Lewis); "A Handbook of Ophthalmic Science and Practice," by H. E. Juler, F.R C.S. (Smith, Elder, and Co.) new edition.

Diseases of the Ear.—"Diseases of the Ear, Throat, and Nose," edited by Dr. Burnett (Lewis); "A Text Book of the Diseases of the Ear," from the German of Professor Josef Gruber (Lewis); "On Diseases and Injuries of the Ear," by Sir William B. Dalby, fourth edition (J. and A. Churchill); "Aids to Otology" ("Aids" series—Bailliere, Tindall, and Cox).

Anæsthetics.—"Anæsthetics, their Uses and Administration," by Dr. Dudley W. Buxton, second edition (Lewis's Practical series); "Anæsthetics and their Administration," by Frederic Hewitt, M.D. (Griffin and Co.)

Practice of Medicine.—"Elements of Practical Medicine," by Dr. Alfred H. Carter, sixth edition (Lewis); "A Manual of Medical Treatment, or Clinical Therapeutics," by J. Burney Yeo, M.D. (Cassells); "Auscultation and Percussion," by Samuel Gee, M.D. (Smith, Elder); "Diseases of the Lungs and Pleuræ, including Consumption," by R. Douglas Powell, M.D. (Lewis); "A Guide to the Examination of the Urine," by H. Lewis Jones, M.D. (Lewis); "What to do in Cases of Poisoning," by William Murrell, M.D. (Lewis).

Diseases of the Skin.—"Diseases of the Skin: Their Description, Pathology, Diagnosis, and Treatment," by Dr. H. R. Crocker, University College, second edition (Lewis); "Diseases of the Skin," by Malcolm Morris, F.R.C.S. (Cassell and Co.)

Midwifery and Diseases of Women.—"A Treatise on the Science and Practice of Midwifery," by W. S. Playfair, M.D., new edition (Smith, Elder); "A Practical Handbook of Midwifery," by Francis W. Nicol Haultain, M.D. (Scientific Press); "Outlines of the Diseases of Women," by John Phillips, M.D. (Griffin and Co.); "A Practical Handbook of the Diseases of Women," by Arthur H. N. Lewers, M.D., fourth edition (Lewis's Practical series).

Diseases of Children.—"The Diseases of Childhood (Medical);" by H. Bryan Donkin, M.D. (Griffin); "Aids to Paediatrics" ("Aids" series, Bailliere, Tindall, and Cox).

Forensic Medicine.—"Forensic Medicine and Toxicology," by J. Dixon Mann, M.D. (Griffin and Co.)

*For continuation of Students' Books, see Nursing Mirror, p. ccxlix.*

